# Immune thrombocytopenia purpura flare post COVID-19 vaccine

**DOI:** 10.1016/j.amsu.2021.103164

**Published:** 2021-12-06

**Authors:** Elrazi Ali, Qusai Al-Maharmeh, Waail Mohammed Rozi, Mhd Baraa Habib, Ahmed Abdallah, Mohammed Abdulgayoom, Mohamed Yassin

**Affiliations:** a Internal Medicine Department, Hamad Medical Corporation, Doha, Qatar; b Nephrology Department, Hamad Medical Corporation, Doha, Qatar; c Department of Hematology and Medical Oncology, Hamad Medical Corporation, Doha, Qatar

**Keywords:** SARS-CoV-2, COVID-19, Immune thrombocytopenic purpura, Vaccine

## Abstract

**Introduction and importance:**

Coronavirus disease 2019 (COVID-19) is a recently discovered disease that has yet to be thoroughly described. It is caused by severe acute respiratory syndrome coronavirus 2 (SARS-CoV-2), a novel virus that can be transmitted easily from human to human, mainly by the respiratory route. The disease often presents with non-specific symptoms such as fever, headache, and fatigue, accompanied by respiratory symptoms (e.g., cough and dyspnea) and other systemic involvement. Currently, vaccination is the primary strategy to prevent transmission and reduce disease severity. However, vaccines have side effects, and the consequences of vaccination in different diseases are not well established. Moreover, the impact of SARS-CoV-2 vaccination during pregnancy is another not well-known area.

**Case presentation:**

We present a young lady known to have ITP, which was controlled for years, presented with relapse after taking the SARS-CoV-2 vaccine during pregnancy.

**Clinical discussion:**

The patient had a relapse of ITP after the introduction of the first dose of the COVID-19 vaccine, which worsened further after the second dose. This suggests that patients with ITP who develop flare post-SARS-CoV-2 vaccine should have their second dose delayed, particularly if pregnant.

**Conclusion:**

To avoid further deterioration in platelet count, and avoid confusion due to the presence of different causes of thrombocytopenia and avoid complications related to thrombocytopenia during pregnancy which can affect the mode of delivery.

**The case is reported in line with the scare 2020 criteria:**

Agha RA, Franchi T, Sohrabi C, Mathew G, for the SCARE Group. The SCARE 2020 Guideline: Updating Consensus Surgical CAse REport (SCARE) Guidelines, International Journal of Surgery 2020; 84:226–230.

## Highlights

• Patients with ITP flare post-SARS-CoV-2 vaccine should have their second dose delayed, particularly if pregnant.
• This will avoid further deterioration in platelet count, which can occur after the second dose of the vaccine and subsequent complications.
• Delaying the second dose of vaccine avoids the confusion about the thrombocytopenia caused during pregnancy which also affects the mode of delivery.

## 1 Background

Severe acute respiratory syndrome coronavirus 2 (SARS-CoV-2) is a novel coronavirus, was identified as the source of a cluster of pneumonia cases in Wuhan, Hubei Province, China, near the end of 2019. It quickly spread over the world, resulting in a global epidemic. The disease severity is more pronounced in patients with comorbidities [1,2]. The presentation of SARS-CoV-2 infection is mainly with respiratory symptoms, but other major organ involvement liver, kidney, and pancreas are also seen [3,4]. Vaccines are the main preventive measures for containing the SARS-CoV-2 epidemic. At the moment, several vaccinations will be available for use in various world regions by the end of 2021. Post-vaccination local and systemic adverse effects are relatively common, particularly after the second dose; most are mild or moderate in severity (i.e., do not prevent daily activities) and are limited to the first two days after vaccination [5,6]. One of the uncommon side effects of vaccination is Immune Thrombocytopenic Purpura (ITP). Primary Immune Thrombocytopenic Purpura (ITP) is an autoimmune disorder characterized by increased platelet destruction and decreased platelet production. The incidence of ITP is around 6 per 100,000 adults/year [7]. ITP has been reported as a result of viral infections or immunizations administered to prevent infectious diseases as in MMR and pneumococcal vaccines [8,9]. After the introduction of SARS-CoV-2 vaccines, thrombocytopenia has been documented in a small number of patients, mainly with adenoviral vector-based vaccines [10]. SARS-CoV-2 vaccinations have been given to millions of individuals only a small, but growing number of cases of “immune thrombocytopenia” or “thrombocytopenia” have been reported to the FDA's VAERS system (10). Based on the available data, post-SARS-CoV-2 vaccination-related thrombocytopenia appears to have a wide range of onset, severity, and duration.

We are reporting a case of Immune Thrombocytopenic Purpura relapse after the two doses of the Pfizer SARS-CoV-2 vaccine during pregnancy.

## 2 Case presentation

**Fig. 1:**
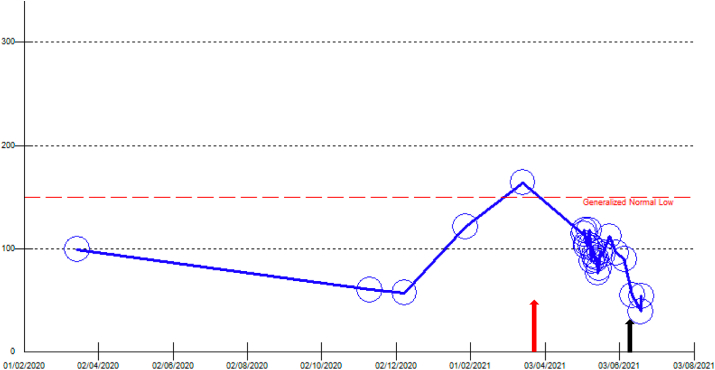
The platelet count trend before and after the vaccination. The red arrow indicates the time of the Pfizer vaccine administration. The black arrow indicates the time of the second dose of Pfizer vaccine administration. (For interpretation of the references to colour in this figure legend, the reader is referred to the Web version of this article.)

A 31-year-old gravida 2, para 1 lady in her 8th week of pregnancy, presented with fever and dry cough for eight days. She has a past medical history of Immune thrombocytopenic purpura (ITP), diagnosed in 2015, fibromyalgia, and hyperthyroidism. She received the first dose of the Pfizer SARS-CoV2 vaccine; fifteen days before the vaccination, the patient had a platelet count of 164 x 10^9^/L. Twenty-six days after the SARS-CoV-2 vaccine administration, she experienced fever and dry cough. She presented to a local emergency department with a fever, and she was admitted. During the admission, she was investigated for fever, and no apparent cause was found. On admission day (post-vaccination day 35), physical examination showed a single palpable painful cervical lymph node in the left anterior cervical lymph node group, and her platelet count was 114 x 10^9^/L. During hospitalization, she received ceftriaxone (2g/day) for three days and azithromycin (250mg/day) for 3 days. Chest x-ray and investigations (Table 1) were within normal limits, including blood cultures, urine culture, flow cytometry, quantiferon, acid-fast bacilli smear, and culture of the sputum and Lumber puncture. Subsequently, subsided alone ten days after the admission day. Additionally, abdominal ultrasound showed a single viable intrauterine fetus and trace of fluid in the right sub-hepatic region. Her platelet count was trending down during the hospital stay, and on the day of discharge, it was 54x 10^9^/L (post-vaccination day 45) (see Fig. 1). The patient was discharged and followed as an outpatient. On June 9, 2021, she received the second dose of the Pfizer SARS-CoV2 vaccine. After two months of follow-up, she was doing well, and platelet readings dropped to 20x 10^9^/L with no major bleeding.

**Table 1:** The table shows the blood investigation done for the patient during admission.

parameter	Result	Normal range
White blood cells	3.3 x10^3/uL	4–10 x10^3/uL
Hemoglobin	11.8 gm/dL	12-15 gm/dL
Platelets	114 x10^3/uL	150–400 x10^3/uL
C-reactive protein	9 mg/L	0–5 mg/L
Creatinine	54 μmol/L	62–106 μmol/L
Urea	3.8 mmol/L	2.8–8.1 mmol/L
Albumin	34 gm/L	35–52 gm/L
Quantiferon TB	Negative	
Peripheral smear	Negative for malaria	
Brucella IgG/IGM	Negative	
Treponema pallidum Ab	Non-reactive	
CMV AB IgG	positive	
CMV AB IgG	Negative	
EPV capsid antigen IgG	positive	
EPV capsid antigen IgM	negative	
ALL respiratory viral pan including COVID-19	negative	
Hepatitis B surface antigen	negative	
Hepatitis c antibody	negative	
Human immunodeficiency virus antibody	Negative	
Tuberculosis PCR	negative	
Acid fast bacilli smear	negative	
Cerebrospinal fluid analysis and gram stain	Negative for bacterial, viral or fungal infection	
Legionella urine antigen	NOT detected	
Blood cultures	negative	
Urine culture	Negative	
TSH	74.80 mIU/L	0.4–4.20 mIU/L
Free T4	3.8 pmol/L	11–23.3 pmol/L
Rheumatoid factor	<10 IU/mL	0–14 IU/mL
Anti nuclear AB titer	Positive 1:160	
Anti DsDNA AB	negative	
Anti thyroglobulin Ab	510 IU/mL	0–115 IU/mL
IgG sub 1	6,998 mg/L	3,824–9,286 mg/L
IgG sub 2	3,835 mg/L	2,418–7,003 mg/L
IgG sub 3	1,239 mg/L	218–1761 mg/L
IgG sub 4	446 mg/L	39–864 mg/L

Characteristics of blood investigation.

## 3 Discussion and conclusion

Vaccination is the most promising approach for containing the coronavirus disease 2019 (COVID-19) pandemic. The first SARS-CoV-2 vaccine was introduced on December 2, 2020, in the United Kingdom. Since then, many vaccines have been introduced, and vaccination side effects are being reported. One of the side effects is thrombocytopenia. Post vaccine thrombocytopenia (VITT) typically occurs within the first 5–10 days post-vaccination and up to 30 days [11]. VITT is thought to be caused by antibodies against platelet factor 4 (PF4, also called CXCL4). These antibodies are immunoglobulin G molecules (IgGs) that activate platelets leading to thrombocytopenia and also thrombosis [12]. The reported thrombocytopenia following vaccination was mainly seen in patients with ChAdOx1 nCoV-19 (AstraZeneca, University of Oxford, and Serum Institute of India) and Ad26.COV2.S (Janssen; Johnson & Johnson). Less frequently, reported with Pfizer and Moderna, and the reported cases include patients with ITP in remission [[13], [14], [15]]. However, the PIVV main feature is thrombosis, and the thrombocytopenia is usually less symptomatic.

Our patient was previously diagnosed with ITP. Additionally, she had positive Anti-nuclear antibodies, hypothyroidism with positive Anti thyroglobulin Ab and elevated IgG subclass 1; all these findings strongly supports that the patient had an autoimmune disorder besides the ITP. She had ITP flare rather than post-vaccine thrombocytopenia as she has not developed any thrombotic complications. She was pregnant, and thrombocytopenia during pregnancy can have several causes. These include HELLP syndrome, severe preeclampsia, Thrombotic cytopenic purpura, and DIC. However, the gestational age and blood investigations did not support these results, as shown in (Table 1).

The main type of Ab in ITP is IgG, mostly directed against platelet membrane glycoproteins such as GPIIb/IIIa [16]. On the other hand, the main target for the post-vaccine thrombocytopenia is the major antigenic target for both SARS-CoV-1 and MERS vaccines was the large surface spike protein [17]. It is not known if there is cross-reactivity between the spike protein and the PDF4.

Pfizer vaccine is an mRNA vaccine, and theoretically, they should not pose a risk to the babies [18]. Our patient was in remission for the last 5 years, and when she took the first dose, she felt fatigued and felt of recurrence of ITP; later, the platelets dropped more significantly after the second dose. It is difficult to conclude if this is related to the effect of the vaccine in pregnant ladies or due to the effect of the vaccine on ITP.

There is little data regarding the COVID vaccine effect during pregnancy. Our patient had the two doses of vaccine, which resulted in ITP flare, particularly after the second dose. The drop in the platelets count was more pronounced after the second dose. This strongly suggests that the underlying mechanism is an immunological process boosted by the second dose of the vaccine, which resulted in a higher titer of Ab and subsequent destruction of platelets.

In summary, patients with ITP flare post-SARS-CoV-2 vaccine should have their second dose delayed, particularly if pregnant. This will avoid further deterioration in platelet count, avoid confusion due to the presence of different causes of thrombocytopenia and avoid complications related to thrombocytopenia during pregnancy which can affect the mode of delivery.

## Ethical approval

The case report was approved by the Medical Research Center with MRC-04-21-621.

## Sources of funding

Qatar National library.

## Authors contributions

Elrazi Ali: conceptualization, writing editing, final approval.

Qusai Al-Maharmeh: writing editing, final approval.

Waail Mohammed Rozi: writing editing, final approval.

Mhd Baraa Habib:writing editing, final approval.

Mohamed Yassin: conceptualization,writing, editing, final approval.

## Registration of research studies

Name of the registry:
Unique Identifying number or registration ID:
Hyperlink to your specific registration (must be publicly accessible and will be checked):

## Guarantor

Dr Elrazi Awadelkarim Hamid Ali.

Dr Mohamed A Yassin.

## Consent

Written informed consent was obtained from the patient for publication of this case report and accompanying images. A copy of the written consent is available for review by the Editor-in-Chief of this journal on request.

## Provenance and peer review

Not commissioned, externally peer-reviewed.

The work has been reported in line with the SCARE 2020 criteria [19].

## Declaration of competing interest

All authors have no conflict of interest.
